# The bioinformatics wealth of nations

**DOI:** 10.1093/bioinformatics/btaa132

**Published:** 2020-03-04

**Authors:** Anastasia Chasapi, Vasilis J Promponas, Christos A Ouzounis

**Affiliations:** b1 Biological Computation & Process Lab (BCPL), Chemical Process & Energy Resources Institute (CPERI), Centre for Research & Technology Hellas (CERTH), Thessalonica, GR-57001, Greece; b2 Bioinformatics Research Laboratory, Department of Biological Sciences, University of Cyprus, Nicosia, CY-2109, Cyprus

## 1 Introduction

In bibliometrics, scientific output is typically measured in terms of quantity, e.g. number of publications, or quality, e.g. number of citations (Almeida *et al.*, 2009). For individual researchers, absolute counts are regarded as sufficient, although it is well-known that these numbers may vary per research field (Yang *et al.*, 2012). The *h*-index, the number of *N* publications that have been cited at least *N* times (Hirsch, 2005), has also been shown to vary across scientific disciplines (Lillquist and Green, 2010). Other, more complex metrics have been devised, yet the *h*-index is indeed a widely used measure of academic ‘success’ or impact (Alonso *et al.*, 2009)—despite the fact that the primary metrics on which it depends are the number of publications and citations (Yong, 2014).

To assess the standing of entire countries, similar measures are in use (Kahn, 2018). Numbers of publications, citations and the *h*-index have all been compared across nations, to investigate trends of scientific performance (Thelwall and Fairclough, 2017), identify the focus of research in countries, country groups or world regions (Lin *et al.*, 2018), and monitor growth or decline patterns in research intensity (Jenab, 2016). For countries, normalization with econometric indices such as population size or gross domestic product (GDP) is usually necessary, if one needs to take into account relative, not absolute, performance (May, 1997). For large numbers such as publications or citations, this step is critical (Chasapi *et al.*, 2019); it is less important for the *h*-index, which is a good measure of performance that reflects the impact of an entire country in science (Harzing and Giroud, 2014). The *h*-index can be compared against other measures, or rank-order countries in a comparative manner (Jacsó, 2009). Criticisms related to *h*-index such as its variation across fields, a certain lack of discriminatory power and dependence on self-citation patterns do not really apply to country-level statistics for a specific field, where the above factors are mitigated, rendering it ideal for this type of comparisons (Jacsó, 2009).

## 2 Materials and methods 

To quantify the output of bioinformatics publications across countries, we have obtained numbers of publications and citations and obtained the *h*-index using the Web of Science (WoS) by Clarivate Analytics (formerly ISI Web of Knowledge) and a simple query, ‘bioinformatics’ for ‘all fields’ and ‘country name’ (slightly edited for accuracy) in the ‘address’ field (date: December 31, 2019; WoS Core Collection, across all years 1900-present—full list in Supplementary Table S1). This straightforward (and reproducible) query returns multiple counts for bilateral or multi-lateral collaborations, not affecting the overall picture—as counts are kept high for the top performers and in fact collaborations are taken into account as a real component of total output (King, 2004).

## 3 Results

**Fig. 1.**
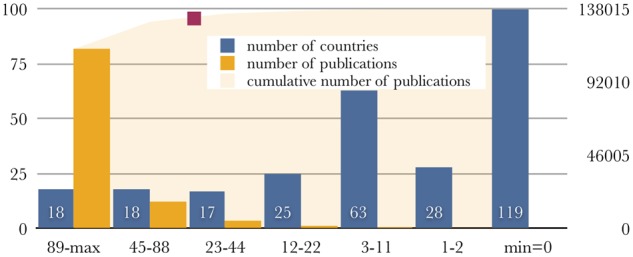
Distribution of number of countries and corresponding bioinformatics publications, for various *h*-index classes. *X*-axis: *h*-index intervals, from high (89-max) to low (minimum=0); *Y*-axis: left, number of countries (blue bars, values shown. clipped at 100) for corresponding *h*-index intervals; right, number of publications (orange bars, values not shown) for corresponding countries (and intervals), cumulative sum of all publications returned by the reported query (orange-shaded surface), amounting to a total of 138 015 publications; the red rectangle signifies the 96% output of the top 40 countries against total (refers to the left *Y*-axis)

We have used a list of 288 countries and territories and queried WoS for publications containing the search terms and requested publications, citations, the citations/publication ratio and the *h*-index for the returned results (four primary indices). The frequency distribution of the *h*-index rank follows an exponential decay curve with the following formula *y* = 152.94e^−0.0312^^*x*^, where *x* is the rank of the entry and *y* is the *h*-index, and *R*^2^ = 0.9812 (Supplementary Table S1). Of the 288 instances, 119 have *h* = 0 and 28 instances have *h* = 1 or 2—these are not further discussed (tiny countries or territories, or scientifically less active). The remaining 141 countries have an *h*-index > 2, 78 of those have an *h*-index > 11, just 53 of them have an *h*-index > 22 and 36 have an *h*-index > 44 (Fig. 1). The least active countries include those in the American, African and Asian tropics, as well as former Soviet republics and parts of the Middle East—unsurprisingly, and consistent with previous findings (Radosevic and Yoruk, 2014). More needs to be done to establish and develop additional activity in these areas, where possible, through international collaboration (Hennemann *et al.*, 2012). Examples of proposed activities and recommendations from our own experience for Greece and Cyprus have been provided elsewhere (Chasapi *et al.*, 2019). Ultimately, the ‘top’ 78 countries generate 137 072/138 015 = 99% of the world’s output in the field of bioinformatics (‘all fields’ in WoS query, as mentioned above).

**Fig. 2.**
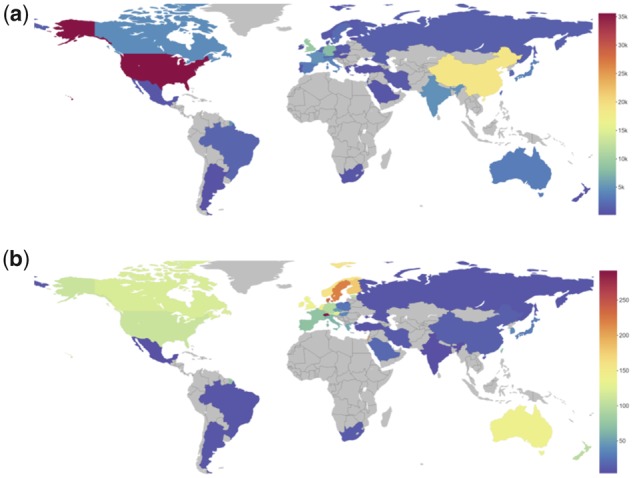
A world map depiction highlighting the top 40 countries in bioinformatics, based on publications output. (**a**) Absolute numbers of bioinformatics publications (scale provided, right), (**b**) relative number of bioinformatics publications per capita (million inhabitants). See Supplementary Table S2 for a full list of 40 countries. Figure generated by Displayr (www.displayr.com)

**Fig. 3.**
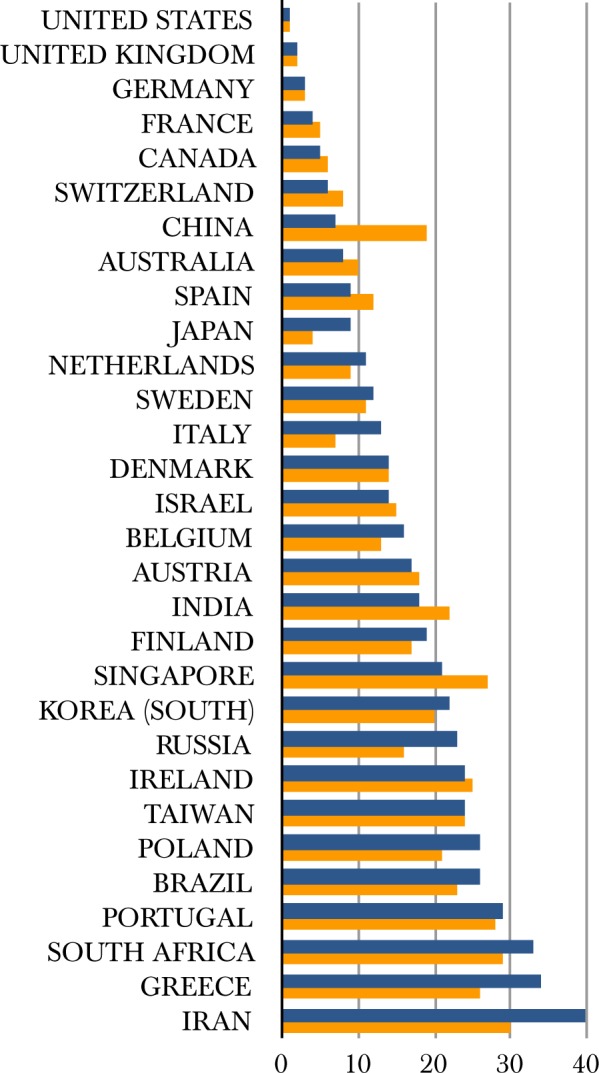
Rank listing of leading countries in bioinformatics and top 1% of highly cited papers. Bioinformatics *h*-index rank (blue), top 1% highly cited papers rank (orange)—lower is better. Thirty countries are listed (see Table 1, and Supplementary Table S2 for a full list of 40 countries)

To examine whether the use of the *h*-index generates a certain bias as a single metric, we have further examined the top 78 countries for numbers of publications in the field and retained only those with >450 publications: this list includes 37 countries, all with an *h*-index ≥ 44 (>10% of the maximum: USA, *h*-index 427), with the exception of Iran (926 publications, *h*-index 37). We have also included three other entries in this list, namely Argentina (420 publications, *h*-index 44), Estonia (133 publications, *h*-index 44) and Hungary (350 publications, *h*-index 52) on the basis of their *h*-index performance (Fig. 2a). Interestingly, when a relative metric such as publications/million inhabitants is used, the resulting picture is slightly different promoting smaller countries with high performance in terms of the number of publications per capita, such as Switzerland or Denmark (Fig. 2b, for details please refer to Supplementary Table S2). The *h*-index ranks of those can be examined in comparison to a group of 30 countries that produce >98% of the world’s highly cited (top 1%) papers (EU15, before 2004 accession and the G8 group, 31 in total, EU excluded here) (King, 2004) and two derived, population-normalized indices (publications and *h*-index per million inhabitants) (Supplementary Table S2). These 40 ‘top’-producer countries generate 132 244/138 015 = 96% of all publications in bioinformatics, according to the WoS query (*cf*. 99% for the 78 countries, above; the remaining 38 have generated just 4828 publications, i.e. 3% of total, Fig. 1). The *h*-index ranking for bioinformatics against the ranking for the 1997–2001 contributions of the top 1% highly cited publications—arguably two independently produced sets—exhibits an astonishing similarity (Fig. 3). The rank (Spearman’s rho) correlation coefficient for the two indices is 0.914 (*P*-value = 0), climbing to 0.964 if Greece, Iran, Italy and Russia are excluded (*h*-index minus top 1% rank difference > 5, Fig. 3). Only Luxembourg (*h* = 27, in the top 1% list: rank 31) is missing (Table 1). Disparities between the two types of rankings may indeed arise from the significant impact of bioinformatics (Wren, 2016). It is worth noting that the ‘elite’ top 1% group has not changed significantly in the past 20 years, as reported recently (Bornmann *et al.*, 2018). The correlation between the ranking of countries with the top 1% cited publications and the country *h*-index for bioinformatics suggests that the leading nations in science with the highest influence and impact in general are, by and large, also those most active in a highly specialized field such as bioinformatics, an expected yet hitherto unknown fact. Our findings also imply that much of the production in the field is generated by the most wealthy nations (GDP or GDP per capita, not shown), raising questions about barriers to entry, and despite a wealth of opportunities for international collaboration, that will need to be addressed in the future.

**Table 1.** List of 30 countries with significant impact in bioinformatics and highly cited publications

Country	*h*-index	rank *h*-index	top 1% rank
United States	427	1	1
United Kingdom	273	2	2
Germany	233	3	3
France	187	4	5
Canada	174	5	6
Switzerland	162	6	8
China	160	7	19
Australia	142	8	10
Spain	139	9	12
Japan	139	9	4
Netherlands	136	11	9
Sweden	129	12	11
Italy	127	13	7
Denmark	120	14	14
Israel	120	14	15
Belgium	116	16	13
Austria	103	17	18
India	93	18	22
Finland	87	19	17
Singapore	82	21	27
Korea (South)	78	22	20
Russia	72	23	16
Ireland	71	24	25
Taiwan	71	24	24
Poland	65	26	21
Brazil	65	26	23
Portugal	58	29	28
South Africa	54	33	29
Greece	53	34	26
Iran	37	40	30

*Note*: Country: country name; *h*-index: *h*-index for bioinformatics (as obtained herein), rank *h*-index: the rank of the *h*-index and top 1% rank: the rank of the country for the world’s highly cited (top 1%) papers. Ranks for the latter are available only for 30 countries, thus the selection of those out of the top 40 countries (Supplementary Table S2).

As the field of bioinformatics has expanded across all of the life sciences (Ouzounis, 2012), the present analysis can form a basis upon which targeted policies for global research and training programs can be implemented, enhancing the productivity of lagging countries to align with the global activity elsewhere, where possible. Such policies might be formulated in alignment with sustainable development goals to match national priorities and perceived public views (Bain *et al.*, 2019), while at the same time maintaining an appropriate balance between global trends and local needs (El-Chichakli *et al.*, 2016).

## Supplementary Material

btaa132_Supplementary_Data
